# High dose insulin therapy for inotropic support during veno-arterial extracorporeal membrane oxygenation decannulation: A case report

**DOI:** 10.1097/MD.0000000000030267

**Published:** 2022-08-26

**Authors:** Kartik R. Shah, Thomas M. Przybysz, Deepu Ushakumari, Ann-Jeannette Geib

**Affiliations:** a Division of Medical Toxicology, Department of Emergency Medicine, Atrium Health’s Carolinas Medical Center, Charlotte, North Carolina, USA; b Department of Pulmonary and Critical Care, Atrium Health’s Carolinas Medical Center, Charlotte, North Carolina, USA; c Department of Anesthesiology, Atrium Health Central Division, Atrium Health’s Carolinas Medical Center, Charlotte, North Carolina, USA.

**Keywords:** cardiogenic shock, extracorporeal membrane oxygenation, glucose-insulin-potassium cardioplegic solution, high dose insulin

## Abstract

High-dose insulin (HDI) therapy has been used as inotropic support for toxin-induced cardiogenic shock, but literature suggests that it can also be used in non-toxin-induced cardiogenic shock states. Its use has not been reported in veno-arterial extracorporeal membrane oxygenation (VA-ECMO) decannulation.

**Patient concerns:**

A 56-year-old male presented with progressive dyspnea and lower extremity edema without any reported toxic ingestion.

**Diagnosis:**

After left heart catheterization, he was diagnosed with acute biventricular nonischemic cardiac failure that ultimately required VA-ECMO support for 8 days, after which decannulation was planned.

**Interventions:**

During decannulation, he was initiated on HDI therapy via a 1 U/kg regular insulin bolus with 25 g of dextrose and a 1 U/kg/hr insulin infusion.

**Outcomes:**

During the decannulation, he was monitored with transesophageal echocardiography. Initially, left ventricular (LV) ejection fraction (EF) was estimated at 10% to 15%. Transesophageal echocardiography after HDI but prior to decannulation showed LVEF 30% to 40%. Transthoracic echocardiography 3.5 hours after HDI bolus and decannulation revealed normal LV systolic function; LVEF 50% to 55%.

**Lessons:**

While multiple interventions occurred during decannulation, HDI therapy may have assisted in transitioning off ECMO support, and HDI should be investigated as an adjunctive option in future decannulations and other non-toxin-induced cardiogenic shock states.

## 1. Introduction

High-dose insulin (HDI) therapy has been used as inotropic support for toxin-induced cardiogenic shock with great success,^[1]^ but literature suggests that its inotropic effects can be used in non-toxin-induced cardiogenic shock states as well, especially when a dose of at least 1 U/kg/hr is used.^[2,3]^ This is an Atrium institutional review board-approved case report of a patient with acute nonischemic biventricular failure who was supported with veno-arterial extracorporeal membrane oxygenation (VA-ECMO) and was transitioned off ECMO support using HDI therapy.

## 2. Case presentation

A 56-year-old 90-kg man with a past medical history significant for hypertension and alcohol use disorder, no history of diabetes, presented to an outside emergency department with progressive dyspnea and lower extremity edema which he first noticed after riding a bicycle. He was discovered to be in atrial fibrillation with rapid ventricular response. He decompensated after receiving intravenous diltiazem in the emergency department. He required mechanical ventilation for pulmonary edema and was supported with multiple vasopressors for cardiogenic shock (left ventricular [LV] ejection fraction [EF] < 20%). He was taken for left heart catheterization which did not show any obstructive coronary artery disease. Then, he had a percutaneous left ventricular assist device (Impella®) placed via his axillary artery. Femoral VA-ECMO was added because of poor left ventricular assist device flows and the clinical deterioration from biventricular heart failure and pulmonary edema. He was transferred to our hospital with cardiogenic shock and multisystem organ failure for further management.

After 8 days of this mechanical and extracorporeal support, including continuous renal replacement therapy, he developed bleeding around the ECMO cannulas and ipsilateral leg ischemia. He was felt to be as optimized as possible for decannulation of VA-ECMO given the new complications and multiple attempts at different vasopressor doses and weaning trials. To provide the most controlled setting and the maximum physician and nurse to patient ratio, toxicology was consulted to use HDI intraoperatively as an adjunctive inotropic therapy during decannulation. The day prior to decannulation, transthoracic echocardiography (TTE) revealed normal LV cavity size and wall thickness with severely reduced systolic function and an EF of <10% during an ECMO wean down to 1 L/min, with maximum Impella® support (P-9 setting, indicating 33,000 rpm). Mild to moderate right ventricular (RV) systolic dysfunction along with severe global hypokinesis were noted. The lowest blood glucose he had in the prior 7 days was 120 mg/dL, and in the prior 5 days, 160 mg/dL. The day before decannulation, his blood glucose was 250 mg/dL, and the morning prior to decannulation, his glucose was 227 mg/dL.

**Figure 1.**
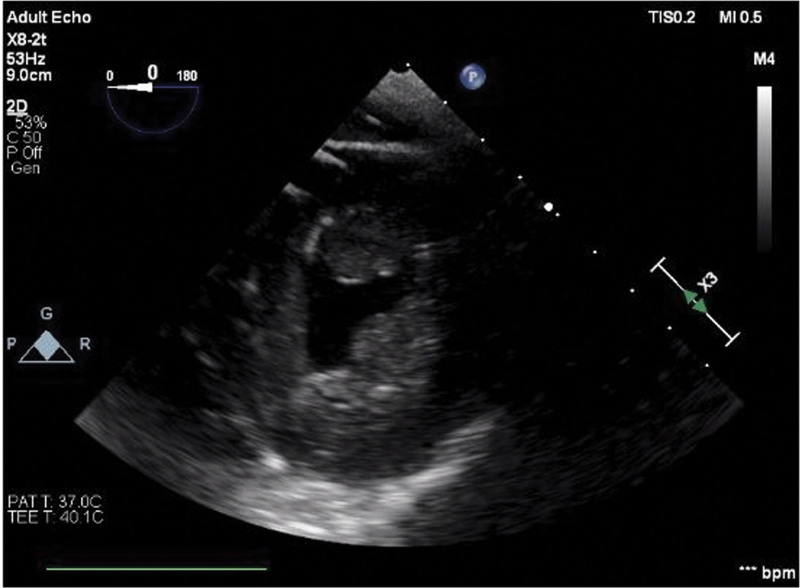
Transgastric short axis view of left ventricle, start of systole, pre-HDI. HDI = high dose insulin.

**Figure 2.**
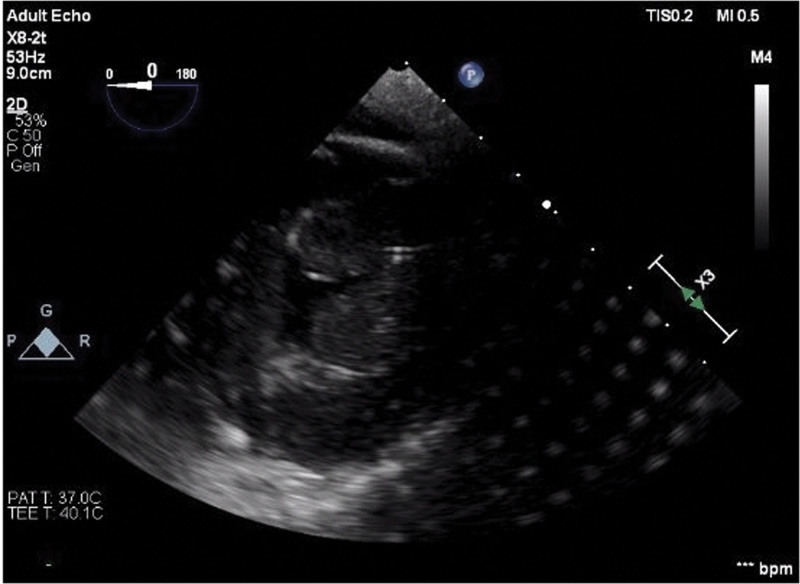
Transgastric short axis view of left ventricle, end-systole, pre-HDI. HDI = high dose insulin.

Intra-operatively during the decannulation, he was monitored with real-time transesophageal echocardiography (TEE). At the start of the procedure, severe biventricular dysfunction was present, with LVEF visually estimated at 10% to 15% while he was on 14 μg/min of norepinephrine and 3 μg/min of epinephrine. Figures 1 and 2 (a clip is available as Supplementary Video S1, Supplemental Digital Content 1, http://links.lww.com/MD/H110) show LV function at start and end of systole in a transgastric short axis view. A 1 U/kg regular insulin bolus with 25 g of dextrose was given 20 minutes prior to decannulation, and a 1 U/kg/hr insulin infusion was started. Repeat TEE prior to decannulation showed LV function of 30% to 40% with significantly improved RV function. During the procedure, he received 3 units of packed red blood cells to replace the volume lost in the ECMO circuit on decannulation. His hemoglobin was 10.6 g/dL 2 hours prior to decannulation and 11.5 g/dL 1 hour after decannulation. After decannulation, with mean arterial pressure 60 to 70 mm Hg, his pulse oximetry started trending down with a nadir of 60% despite optimizing ventilatory settings (100% fraction of inspired oxygen and positive end-expiratory pressure of 14 mm Hg) as well as a trial of an inverse ventilation ratio mode. An arterial blood gas revealed a pH of 7.23, pCO2 of 59 mm Hg, and pO2 of 39 mm Hg. A bolus dose of milrinone at 50 μg/kg was administered. This was followed with inhaled nitric oxide to decrease pulmonary vascular resistance, vasopressin infusion to counteract the systemic vasodilation from the milrinone, and increased epinephrine and norepinephrine infusions to 8 and 14 μg/min, respectively. SpO2 improved to 100% 15 minutes after the bolus dose of milrinone. PA pressure before decannulation was 5 mm Hg while after these interventions, it was 35 mm Hg. Milrinone infusion was started at 0.25 μg/kg/hr. The patient was weaned down from 8 μg/min of epinephrine to 4 μg/min and 14 μg/min of norepinephrine to 8 μg/min.

**Figure 3.**
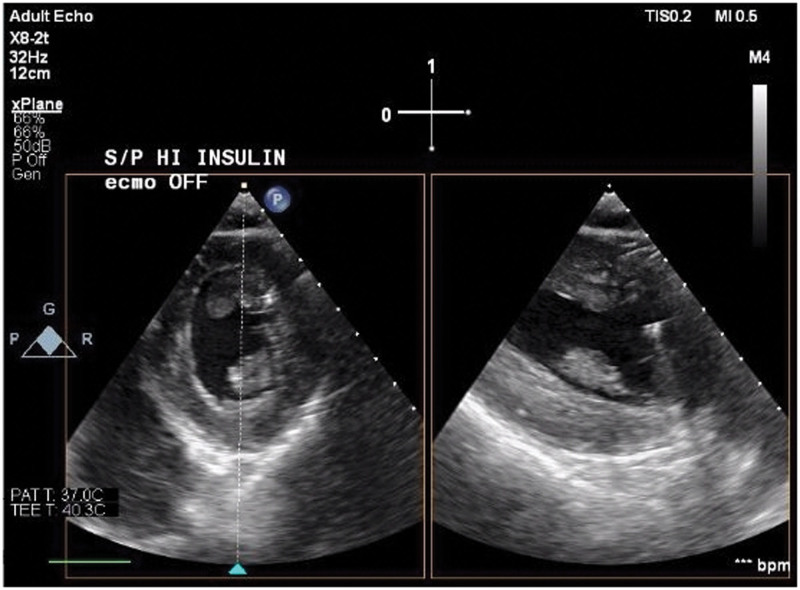
Transgastric short axis view of left ventricle, start of systole, post-HDI, and decannulation of ECMO catheters. ECMO = extracorporeal membrane oxygenation, HDI = high dose insulin.

**Figure 4.**
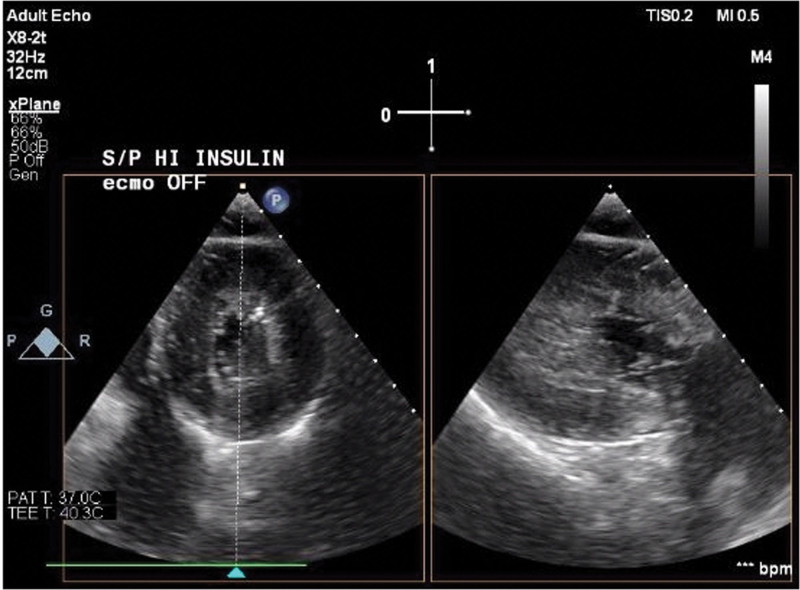
Transgastric short axis view of left ventricle, end-systole, post-HDI, and decannulation of ECMO catheters. ECMO = extracorporeal membrane oxygenation, HDI = high dose insulin.

Repeat TEE showed persistently improved LV function (Figs. 3 and 4; a clip is available as Supplementary Video S2, Supplemental Digital Content 2, http://links.lww.com/MD/H111), so epinephrine was stopped due to episodes of supraventricular tachycardia. The milrinone infusion was subsequently stopped and the norepinephrine was increased in the ICU. TTE 3.5 hours after HDI bolus, when he was on 1 U/kg/hr of HDI, 18 μg/min of norepinephrine, 0.04 U of vasopressin, and inhaled nitric oxide, revealed normal LV systolic function with an EF of 50% to 55% and mildly reduced RV systolic function. HDI infusion was continued for 24 hours after the bolus. Initial glucose after the dextrose bolus was 488 mg/dL. A dextrose infusion was started 5 hours after HDI bolus, titrated for a blood glucose >150 mg/dL, with rates of 0.33 to 0.5 g/kg/hr. His potassium was 3.6 mEq/L before HDI bolus, and his nadir was 3.2 mEq/L 12 hours after the HDI bolus. He received only 1 dose of 30 mmol/L of potassium-phosphate in total exogenous potassium before insulin infusion was stopped. After the insulin infusion was stopped, the dextrose infusion continued for 24 hours, and an additional 30 mmol/L of potassium-phosphate was given. His glucose the day after decannulation was 181 mg/dL.

The patient continued to progress over the next several weeks, with extubation 6 days later. A hemoglobin A1C 21 days after decannulation was 5.2%. Ultimately, he was discharged home 26 days after ECMO decannulation with outpatient therapies arranged with a diagnosis of dilated non-ischemic cardiomyopathy without a confirmed etiology. On cardiology outpatient clinic follow-up 2 weeks later, he was alert and oriented with no focal neurologic deficits. He had a repeat TTE 6 months later which showed LVEF of 15% to 20%, severely reduced RVEF, and global hypokinesis but had resumed activities, including cycling 10 miles a day outdoors.

## 3. Discussion

There is a wide literature base surrounding the use of insulin for inotropic support, with varying terminology used, such as glucose-insulin-potassium (GIK) therapy, hyperinsulinemia euglycemic therapy, and HDI therapy. Insulin’s inotropic effects are thought to be multifactorial and are not fully elucidated. One common mechanism suggested is enhanced myocardial substrate handling. In a nonstressed state, the myocardium primarily uses free fatty acids, but in a stressed state, it relies more on carbohydrates. Insulin is thought to support the myocardium during this change in metabolism.^[4,5]^ However, there is evidence that other mechanisms also play a role in the increased inotropy.^[6]^ These include effects on calcium, potassium, and sodium ion balance.^[7]^ An additional mechanism that has not been fully explored is insulin’s effect on protein kinase B, aka Akt.^[8,9]^

Studies evaluating insulin’s effects have mixed results when viewed altogether. A systematic review and meta-analysis found no mortality benefit for GIK in critically ill patients^[10]^; however, this analysis was heavily weighted by the CREATE-ECLA study.^[11]^ CREATE-ECLA was a randomized placebo-controlled trial of GIK in over 20,000 patients having acute myocardial infarction, but 68.3% were given GIK after reperfusion therapy had already been administered. Moreover, the doses in all but one of the studies (Koskenkari et al, discussed below) of the aforementioned meta-analysis, including CREATE-ECLA, are close to 0.1 U/kg/hr, an order of magnitude less than the 1 U/kg/hr rate that toxicologists routinely use in their treatment of toxin-induced cardiogenic shock.^[1]^ As such, we differentiate insulin infusions at a rate of 1 U/kg/hr or more as HDI instead of GIK, and we believe it is critical that the literature must be evaluated with this key, important difference of dosing in mind; hyperinsulinemia euglycemic therapy is an older term that has fallen out of favor as it can be misconstrued as suggesting the goal of the therapy is glucose control instead of inotropic support and improved perfusion, which often need exogenous glucose to be administered to be safely obtained.

Koskenkari et al^[2]^ performed a placebo-controlled RCT of HDI at 1 U/kg/hr in patients scheduled for combined aortic valve replacement and coronary artery bypass surgery and found that HDI led to better preserved myocardial contractile function and less need for inotropic support with dobutamine. In another RCT, Hiesmayr et al^[3]^ found that in patients undergoing coronary artery bypass grafting, both HDI at 1.5 U/kg/hr and dobutamine 7 μg/kg/hr increased cardiac performance, but HDI did not increase myocardial oxygen demand.

Animal studies in non-toxin-induced cardiogenic shock also support the use of HDI. One of the early studies using HDI involved a canine acute ischemic cardiogenic shock model created by placing beads in the canines’ left main coronary arteries. A HDI bolus caused a dramatic improvement in cardiac output.^[12]^ Similarly, in a porcine model of septic shock created by infusing *Escherichia coli* endotoxin lipopolysaccharide, HDI improved cardiac function.^[13]^ Uncontrolled human studies also show improved cardiac function in sepsis after HDI therapy.^[14–17]^

HDI is not without its drawbacks. It can cause metabolic derangements, in particular hypoglycemia and hypokalemia, though data suggest there is a ceiling to the magnitude of these effects.^[18]^ Serial monitoring is required for these effects, including recommended blood glucose checks every 30 minutes. Our patient needed a dextrose infusion for 24 hours after insulin cessation, which is common in the authors’ experience in using HDI in toxin-induced cardiogenic shock. It was unusual that it took several hours for the dextrose requirements to fully manifest however. The fairly minor amount of hypokalemia seen was also surprising; perhaps the exogenous and endogenous beta agonism had already led to an intracellular shift in the potassium, or the continuous renal replacement therapy was modulating the electrolyte shift. As the hypokalemia is merely an intracellular shift, many toxicology HDI protocols do not provide any potassium until the serum potassium is below 2.5 mEq/L, though as our patient had arrhythmias already, a higher serum potassium was likely beneficial.

Overall, as with any case report, there are many confounding factors for the patient’s clinical course, such as the multiple interventions performed during ECMO decannulation and visual estimations of LVEF on TEE instead of quantitative measurements. However, our patient had an impressive response to HDI therapy during real-time TEE performed and interpreted by our anesthesiologist who has decades of experience (author DU), with a normal EF on TTE shortly after HDI initiation and grossly depressed EF on TTE the day prior and 6 months later on outpatient follow-up. We believe HDI at this dose likely assisted the patient in transitioning off of ECMO support and should be investigated as an adjunctive option in future decannulations and other non-toxin-induced cardiogenic shock states.

## Author contributions

KS wrote the manuscript. All authors treated the patient, revised the manuscript, and have reviewed and approved the final draft.

**Investigation:** Ann-Jeannette Geib, Deepu Ushakumari, Kartik R. Shah, Thomas M. Przybysz.

**Methodology:** Ann-Jeannette Geib, Deepu Ushakumari, Kartik R. Shah, Thomas M. Przybysz.

**Supervision:** Ann-Jeannette Geib, Deepu Ushakumari, Kartik R. Shah, Thomas M. Przybysz.

**Writing – original draft:** Kartik R. Shah.

**Writing – review & editing:** Ann-Jeannette Geib, Deepu Ushakumari, Kartik R. Shah, Thomas M. Przybysz.

## Supplementary Material


